# Challenges and facilitators to perinatal mental healthcare among first-generation migrant women: A qualitative ethnographic study in Flanders, Belgium

**DOI:** 10.18332/ejm/194682

**Published:** 2024-12-02

**Authors:** Astrid Claerbout, Jade Steppe, Gilissen Joni, Liesbeth van kelst

**Affiliations:** 1 Research Centre Care in Connection, Karel de Grote University of Applied Sciences and Arts, Antwerp, Belgium; 2 Department of Family Medicine and Chronic Care, Vrije Universiteit Brussel, Brussels, Belgium; 3 Department of Public Health and Primary Care, Universiteit Gent, Ghent, Belgium

**Keywords:** mental health, health literacy, immigration, perinatal care

## Abstract

**INTRODUCTION:**

Women with a migration background face significant barriers to accessing perinatal mental health support. This study aims to explore the needs, barriers and facilitators regarding perinatal mental health support in women with a first-generation migration background and how they experience support within their own community.

**METHODS:**

We conducted qualitative in-depth face-to-face interviews with mothers who gave birth within 12 months preceding the interview, recruited from an Antwerp maternity ward between August and September 2022. Three midwife researchers conducted interviews at the participants' home, using an interpreter if needed. A midwife researcher with relevant expertise reviewed the final manuscript. Two researchers independently performed qualitative inductive content analysis and constant comparison of audio transcripts using NVIVO.

**RESULTS:**

Of the 11 participants, none reported mental health issues at the time of the interview. However, four mothers shared experiences indicative of postnatal depression, highlighting significant challenges such as isolation, language barriers, and a lack of awareness about mental health support. Recurring themes included the importance of professionals taking the time and making efforts to provide accessible information, navigating language barriers, differences in participants' openness toward discussing mental health, the importance of having a close network available for psychological support, and practical aspects inhibiting access. Overall, participants reported a desire for more culturally sensitive care and information about available support.

**CONCLUSIONS:**

Findings underscore the urgent need for tailored perinatal mental health support that is easy to access, emphasizing awareness and training for professionals, especially midwives, to meet diverse needs. Recognizing this population's variety is essential.

## INTRODUCTION

In the European region, the presence of international migrants in the population is not only substantial but also on the rise, reaching nearly 10% of the total population^1^. A significant portion of these migrants comprises women of reproductive age. Consequently, European maternity care systems and professionals are increasingly likely to face the challenge of delivering high-quality healthcare specifically tailored to meet the unique needs of women with a migrant background^2-4^.

Although there is heterogeneity in the degree to which immigrants are vulnerable to inadequate healthcare, they have been identified as a generally vulnerable population^5^. These differences are more complicated by pregnancy^6,7^. Subsequently, specific attention should be paid to pregnant women with immigration backgrounds during perinatal care^8^.

Although immigrant women, constituting a significant and diverse population, often face unique challenges during this crucial period, and variations exist in perinatal mental health across different ethnic groups. Overall, migrant women seem to face inadequate pregnancy follow-up, resulting more often in adverse mother and neonatal outcomes, and the women are more likely to face mental health issues^8^, have more miscarriages, fetal growth problems, premature birth, and high perinatal mortality^7,9,10^. Subsequently, perinatal mental health conditions directly impact the child’s behavioral, cognitive, social and emotional development and disrupt maternal–infant attachment and family functioning^11^. Barriers to accessing healthcare, cultural differences, language barriers, and knowledge of the common healthcare practices in their new residence, are often reported causes for multifactorial healthcare inequality and injustice^12,13^. In refugees and asylum seekers, these inequities are worse^12^.

Perinatal mental disorders are common – the most common complication of child-bearing – and are associated with considerable maternal and fetal/infant morbidity and mortality^8^. Perinatal mental health issues among immigrant women are common^6^ and the mentioned challenges may be particularly true for first-generation migrants. Especially because of pre-migration stressors such as conflict, transition stressors including poverty, and post-migration stressors such as navigating the immigration system. These stressors can make them vulnerable to mental illness^14^. The global prevalence of perinatal depressive disorders among women who have a migrant background is estimated to be between 19% and 31%, while in the overall population of perinatal women, the prevalence is estimated to be 12–17% (for depression)^14^. There is consistent evidence of reduced identification and management of perinatal mental health for ethnic minority women, both for those who do or do not speak English^13^. Due to social COVID-19 restrictions, physical appointments often could not take place, leading to an increased reliance on online alternatives, exacerbating the gap between different communities with an increased likelihood of perinatal mental health challenges^15^.

The existing literature, however, has predominantly focused on quantitative measures of perinatal care utilization and outcomes among immigrant populations^7^ or satisfaction and attributing factors^16^. For Flanders specifically (where the larger proportion of Belgians live, 6.5 million inhabitants), there are no statistics on mental health and the perinatal period among migrant women, and there is only anecdotal knowledge about the needs and barriers women with an immigrant background face. Hence, the perspectives of first-time immigrant women on perinatal care in Belgium remain underexplored, particularly in terms of what might help them to access mental health support during perinatal care. This qualitative study aims to explore the barriers and facilitators of women regarding perinatal (mental) support with a first-generation migration background (born in a country other than Belgium), and whether and how they experience support within their own community.

## METHODS

### Research team and reflexivity

The research team comprised three midwives who conducted the interviews. This study was part of the PATH project, funded by Interreg 2 Seas, which aimed to examine perinatal mental health and was conducted from 1 February 2019 to 21 March 2023. As insider researchers with experience in midwifery clinical practice in large hospitals serving women from immigrant backgrounds, personal insights and experiences significantly shaped the research idea. The researchers were mindful of their positionality as non-immigrant, Dutch-speaking professionals and engaged in regular reflexive discussions throughout the study to mitigate potential biases. All researchers received training in qualitative methods. Participants were informed about the researchers’ academic backgrounds, the purpose of the study, and the broader goals of understanding barriers and facilitators in perinatal care among an immigrant population. An interpreter in the participant’s native language was used in 9 of the 11 interviews.

### Study design and setting

We employed a qualitative ethnographic study design including individual in-depth interviews. The study population consisted of mothers residing in Belgium but not born in Belgium (first-generation migrants) who had given birth in the maternity ward of an Antwerp hospital in Flanders, Belgium. The reporting is in line with the Consolidated Criteria for Reporting Qualitative Research checklist (COREQ)^17^.

### Participants

*Eligibility criteria*

Participants were eligible for the study if they met several specific inclusion criteria. They had to be aged ≥18 years and have at least one child who was aged between 6 weeks and one year at the time of the interview. This timeframe was carefully selected to minimize the risk of distortion from the HALO effect^18^, which can occur if interviews are conducted earlier than 6 weeks postpartum, and to avoid recall bias^19^, which may arise if interviews are conducted later than one year postpartum. Additionally, participants were required to have resided in Flanders from the start of their pregnancy, with both the delivery and postpartum period occurring in Flanders. Women were also included in the study if they had limited proficiency in Dutch, French, or English, or if they agreed to have an interpreter present during the interview. In this context, ‘not proficient’ refers to women who require assistance in their native language to fully understand and communicate during healthcare interactions. However, proficiency is considered relative to the situation; if a woman was able to participate in an interview without the help of an interpreter, she may be considered proficient for the purposes of this study, even if her language skills were not fully fluent.

We did not include participants based on the reason for their immigration. Reasons could vary – consistent with what is known in the literature that the underlying reasons for immigration are multifactorial, involving a complex interaction between factors within and beyond individuals’ control including political, socioeconomic, and educational, along with more acute drivers such as natural disasters, violence, and conflict^20^. We also did not include mothers based on having experienced mental health issues or challenges, or having had a formal diagnosis of a perinatal mental disorder. However, we asked mothers how they felt during pregnancy, during the hospital stay, and after giving birth.

*Recruitment and sampling*

Starting June 2022, the researchers specifically approached the recruitment site since they are known for a varied population in their maternity ward. The researchers briefly informed the physicians and head nurses of the maternity ward.

Participants were identified through a key person (the head midwife) who checked daily the patient population on the maternity ward and who was given a checklist containing the eligibility criteria to determine whether a mother was eligible to take part in the research or not. Eligible mothers were informed by the midwife most involved in their care about the study and were asked if the researcher could enter the room to explain (face-to-face) the study, and obtain contact details. A flyer/poster in Dutch, French, and English (Supplementary file Material 1) was used to explain the research, containing a concise and graphically presented summary.

When the mother expressed initial interest in participating in the research, contact information was gathered for a follow-up meeting/contact (at minimum 6 weeks after the first contact) to further (re-)inform the mother about the study (orally). At that stage, an interpreter was made available if perceived necessary. Subsequently, the information sheet was explained verbally in their native language. The interpreter read the written informed consent aloud, after which it could be signed. The interpreter translated the questions posed by the researcher and then translated the participant’s response. Both the researchers and the interpreters signed the confidentiality statement. The study population was purposively selected to obtain a sample with maximum variation concerning the following criteria (maximum variation purposive sampling)^21^: age, mode of delivery, duration of residency in Belgium, and country of birth.

### Data collection

The data were collected through individual face-to-face, in-depth interviews between August and September 2022 by two researchers (AC, JS). The interview took place at a location chosen by the participant at a minimum 6 weeks after giving birth. An interpreter was used if needed. All interviews were conducted in participants’ homes, with half involving the presence of children, family members, or partners. We operated the necessary sensitivity regarding the gender of the interpreter (i.e. a male interpreter was not used if the mother objected).

An interview guide was used, developed through a rapid search of academic literature via PubMed and constructed in close consultation with the Flemish Expertise Network for Perinatal Mental Health (Supplementary file Material 2). The interview guide, including questions and prompts, was iteratively adapted after each interview when new aspects arose. To enhance participant engagement, the themes with corresponding pictograms were displayed, allowing participants to choose the sequence of the themes. We explored sociodemographic factors orally. Interviews took between 1 and 1.5 hours, including research briefing, informed consent, and interview. All interviews were audiorecorded, and field notes were taken to capture non-verbal communication.

### Data analysis

The interviews were digitally recorded and transcribed verbatim by an independent agency. Along with notes on non-verbal communication, this served as the basis for a qualitative inductive content analysis with constant comparison in NVIVO 12.2 software. The number of data coders involved in coding the data was two researchers (AC and LVK), who evenly divided the transcripts of the interviews. In inductive content analysis, categories/themes are derived from the data. The researchers independently applied open coding to their transcripts and then shared their codes to enhance consistency and reliability. During the analysis, the researchers iteratively sought to identify patterns and similarities, and differences in the data. Similar codes were given similar names and grouped into an overarching theme. The themes were derived inductively from the data. To ensure the confirmability and credibility of the findings, self-checks and peer review were conducted, along with participant triangulation through maximum variation sampling to incorporate diverse perspectives. Additionally, data triangulation was achieved by using both in-depth interviews and observations. The researchers consulted within each phase of the study to determine the next step. Together they applied axial coding, looking for patterns that revealed relationships between different themes. The transcripts were not returned to participants, and all themes, including the relationships between them, are described in the results section.

### Definitions

In this study, ‘depression’ follows the same definition as the Diagnostic and Statistical Manual of Mental Disorders, Fifth Edition (DSM-5)^22^: a mood disorder characterized by persistent sadness, loss of interest in daily activities, and a combination of other symptoms such as changes in appetite, sleep disturbances, fatigue, feelings of worthlessness, and difficulty concentrating. In this study no formal testing occurred to form a diagnosis, where at least five of these symptoms must be present for two weeks, with one being either a depressed mood or loss of interest. ‘Mental health’, according to the World Health Organization (WHO)^23^ is defined in this study as not merely the absence of mental illness but a state of well-being in which individuals can realize their potential, cope with normal life stresses, work productively, and contribute to their communities. It encompasses emotional, psychological, and social well-being. The term ‘perinatal mental care’ in this study encompasses any care related to perinatal mental distress and is not restricted to diagnosed mental health issues such as postnatal depression.

### Ethical considerations

All participants received information about the course of the study, considerations regarding privacy and confidentiality of their participation and the data. This research was submitted for approval to the Ethical Advisory Committee for Social and Human Sciences at the University of Antwerp (Approval Number: SHW_22_050 – Approval Date: 25 March 2022). If the interview revealed that mental difficulties did occur recently, explanations were given by the interpreter using the aftercare list carefully developed and provided by the research team (Supplementary file Material 3). The participant was asked whether she could be contacted again by the researchers to check how things were going, and, if needed, to provide additional assistance with navigating them to specialist care or other informal caregivers, if appropriate and desired.

## RESULTS

### Participants

In total, 11 mothers were interviewed (Table 1). Most of the interviews were conducted with the assistance of an interpreter in the participant’s native language. The majority of all participants indicated that war in their home country motivated them to migrate to Belgium between 2 to 5 years before the interview.

**Table 1:** Characteristics of mothers who participated in the qualitative interview study conducted in Flanders, Belgium, 2022 (N=11)

*Characteristics*	*n (%)*
**Interpreter involved**	9 (82)
**Reason for immigration**	
Family reunification	4 (36)
Studies	1 (9)
War	6 (55)
**Country of birth**	
Morocco	3 (27)
Palestine	1 (9)
Syria	2 (18)
Kosovo	1 (9)
Cameroon	1 (9)
Iraq	1 (9)
Paraguay	1 (9)
Russia	1 (9)
**Residency in Belgium**	
<12 months	1 (9)
12–23 months	1 (9)
2–5 years	4 (36)
>5 years	3 (27)
Not specified*	2 (18)
**Age**, median (range)	21 (28–38)
**Mode of delivery**	
Vaginal	9 (82)
Cesarean section	2 (18)
**Family status**	
Single	1 (9)
Has a partner	10 (91)
**Education level**	
Secondary school not completed	5 (54)
Secondary school completed	3 (27)
Higher education started	3 (27)

* Duration of residency in Belgium was not specified.

Most mothers indicated that they were not experiencing mental problems at the time of the interview (not formally diagnosed but self-reported by participant). However, four mothers did indicate they experienced ‘feeling (really) down’ and ‘having a bad period’, referencing postnatal/postpartum depression. We did not formally assess this in our selection and inclusion procss.

We present examples of relevant quotes of the mothers, their country of origin, and duration of residency in Belgium:

*‘It was a short period, nine or ten days, but I did experience it [referring to postnatal depression]. I was really down, I didn’t even leave my room, much less alone the house. I didn’t feel like doing anything, but after that period, I was able to regain my balance and resume my normal life. Going out with the children, spending time with my husband or my kids. Also, the visit from my brother really did me good.’* (Mother 3, Palestine, 1.5 years)

*‘You won’t be able to make phone calls when you feel bad. So, that was a very bad period. I think that was postpartum depression or something, but then it gets better.’* (Mother 4, Syria, 7 years)

*‘My pregnancy was actually very difficult because I hadn’t foreseen that. It happened unexpectedly, and in my culture, it’s not really accepted for a woman to have a child, to get pregnant without being married. So, that was very difficult for me. I thought about suicide.’* (Mother 5, Kosovo, 10 months)

### Barriers and facilitators regarding perinatal care

The following barriers and facilitators emerged that reflected mothers with an immigration background needs and barriers regarding perinatal care (Table 2).

**Table 2:** Summary table of barriers and facilitators to perinatal mental health support mentioned by first-immigrant mothers who participated in a qualitative interview study in Flanders, Belgium, 2022 (N=11)

*Barriers*	*Facilitators*
**Inaccessible information** Lack of time and attention from healthcare professionals. Negative experiences with quick consultations.	**Healthcare provider support** Instances of compassionate care that made mothers feel respected. Positive experiences with attentive healthcare professionals.
**Community support challenges** Language barriers hinder social connections. Cultural differences in availability and willingness to connect.	**Support from partners or family** Reliance on partners for emotional support. Use of digital communication to maintain family connections.
**Language barriers** Limited proficiency in Dutch/French. Instances of uncooperative healthcare staff. Reliance on unvalidated sources on the Internet to retrieve information.	**Cultural openness about mental health** Perception of mental health discussions being more accepted in Belgium. Support from the local community when discussing mental health issues.
**Unsupportive cultural attitudes** Taboos surrounding mental health in home countries. Differences in openness to discussing mental health issues.	**Digital translation** Availability of apps for translation.
**System barriers** Lack of knowledge about available mental health resources. Financial constraints and long waiting lists.	

*Taking the time and making efforts to provide accessible information*

The women expressed the importance of healthcare professionals having sufficient time, patience, and attention, adapting their communication to provide adequate information. They did not specify who. As such, participants indicated they felt ‘treated like a human’ and they felt they were treated with respect. Two women had positive experiences in this regard and spoke warmly about their healthcare provider, while two other women felt that healthcare providers did not want to spend much time with them to examine them or provide information:

*‘Yes, I received all the attention I needed. My GP promptly referred me to the hospital for examinations, and there, doctors and staff were very attentive. Despite some setbacks, they were patient and followed up regularly.’* (Mother 4, Syria, 7 years)

*‘The specialist did not perform a complete examination. That was just the minimum, and he did not spend much time with me. Five minutes. But I noted that for others, he actually took, for example, fifteen minutes.’* (Mother 7, Cameroon, 2 years)

*Difficulties in establishing a new ‘community of support’*

Most women highlighted the difficulties in establishing a support network in Belgium, citing language barriers and current unemployment as limiting factors that hindered their ability to forge new connections. One woman specifically noted her perception that Belgian women appeared to have less time due to their work commitments, a contrast to her own cultural experience where women typically had more time for interpersonal connections:

*‘A very big difference because here, people don’t have time. They work all week. They don’t even have time on weekends because they also have to do groceries, they have tasks. In Syria, women usually don’t work, well, some women study or something, but if you don’t study or work, then you stay at home, then you have time for others, the family. So actually, a big difference.’* (Mother 4, Syria, 7 years)

*‘I didn’t know anyone here. I also have no family here, except for one aunt who lives in Brussels. But she was on vacation in Morocco when I gave birth in the summer.’* (Mother 2, Morocco, 3 years)

*Navigating language barriers*

The vast majority of participants saw language as a significant barrier. Within healthcare, most providers make an effort to communicate in another language (e.g. English, Spanish) so that parents can understand the information. Although most providers generally tried, there are instances where mothers indicated healthcare staff were unwilling to communicate in a language other than their own. And they sometimes felt that their lack of understanding can lead to heightened impatience from others.

In case of a significant language barrier, participants relied on their partners or family members to translate over the phone, or they used translation applications, albeit suboptimal. The presence of interpreters varies, sometimes available and sometimes not.

Lack of proficiency in Dutch or French (primary languages in Belgium) posed challenges, as it hindered mothers’ comprehensive understanding and their ability to ask questions freely:

*‘But because of the language, because I don’t understand Dutch, I am limited. I don’t have information, or not enough information. I noticed that translation apps don’t always work optimally.’* (Mother 7, Cameroon, 2 years)

*‘You are insufficiently informed if you do not speak the language. You cannot understand everything, but also cannot ask everything. When people do not understand you, they sometimes behave impatiently.’* (Mother 10, Paraguay, unspecified duration)

*‘Everyone did their best to communicate with me except one nurse who said, “We are in Belgium, and you must speak Dutch”. I explained that I had been learning Dutch but had to pause due to my pregnancy. She insisted I speak only in Dutch. I said I spoke a little Dutch but not enough to explain my needs. Eventually, another nurse came, and I could communicate with her.’* (Mother 3, Palestine, 1.5 years)

*Cultural differences in attitude or openness toward discussing mental health*

The interviewed women observed a difference in cultural attitudes toward discussing mental health problems in their home countries compared to Belgium. Most women indicated that talking about mental health problems is often difficult or taboo in their home countries (due to a multitude of factors including religion, inaccessible healthcare, or a lack thereof) which discourages them from seeking professional help. They felt as if it was more accepted in Belgium to talk about mental health issues. The latter was welcomed by most:

*‘Yes [there is a taboo to talk about mental health in their country of origin]. If someone goes to a psychologist, they start saying that he is crazy.’* (Mother 10, Iraq, 3 years)

*‘Talking about mental health in Belgium is completely normal. But talking about mental health in Kosovo, people are totally different. It’s a misunderstanding.’* (Mother 5, Kosovo, 10 months)

*‘They also avoid going to a psychiatrist or something because they think, oh, others will say I’m crazy.’* (Mother 6, Syria, 2 years)

*‘The biggest difference is that here the problem is addressed and considered a serious problem. … But in Cameroon, the problem is not acknowledged.’* (Mother 7, Cameroon, 2 years)

*‘Here, people are more open to such topics. They help you in every possible way. It’s a big difference.’* (Mother 10, Paraguay, unspecified duration)

*‘The problem is we have very few mental health practitioners who specialize in mental health. There’s a lack of specialists to address these issues deeply, so many people don’t realize the problem exists. For instance, if you have a mental health issue, you might stay home and even commit suicide without anyone recognizing you had depression. People might think it was caused by something supernatural, like being possessed by the devil.’* (Mother 7, Cameroon, several years)

*Barriers to seeking professional mental health support*

Half of the women mentioned that they did not know where to seek professional help for mental health problems in Belgium. The other half seemed to be well-informed and knew that they could turn to a psychologist or psychiatrist. However, the associated cost and waiting lists for professional help were mentioned to form a significant barrier:

*‘If I experienced mental difficulties, I would search for a shrink, and I would go there. For now, I feel good, but if it was necessary, I would go.’* (Mother 6, Syria, 2 years)

*‘I received a few treatments that stopped, and I wanted to continue, but she said it would cost you so much every month. I said, yes, I can’t afford that.’* (Mother 11, Russia, several years)

Support within own community

All women emphasized the importance of a social network for their mental well-being. Most relied on their partners as their primary source of emotional support. Other family members, despite being far away, also provided crucial support through the Internet and phone. Healthcare providers and neighbors were mentioned as potential support sources, but less frequently and not as a first choice:

*‘So basically, I pick up the phone, and then I call family via the Internet, of course. … Anyway, I don’t have many friends here; we do have my husband’s friend.’* (Mother 3, Palestine, 1.5 years)

*‘I only talk to my family. I don’t have work colleagues. I don’t have friends. Except friends of the family itself. So that’s it.’* (Mother 10, Paraguay, several years)

*‘So, my husband is actually the sole person with whom I can talk best about everything. I tried to keep it to myself the first two days, but then I just couldn’t hold on anymore. And especially the girls saw that in me, or they noticed that too, and then they would come to me and ask: what’s wrong, Mom, talk to us, can we do something for you and so on. And then at a certain moment, I talked to my husband about it, that I do have postnatal depression. So, he also did his best to get me out of it.’* (Mother 3, Palestine, 1.5 years)

## DISCUSSION

The present study sought to explore the barriers and facilitators to perinatal mental health support among first-generation migrant women in Belgium and whether they felt supported within their community. Through indepth interviews, these women’s experiences revealed key insights into their interactions with healthcare professionals and support networks. Participants highlighted several challenges related to perinatal mental healthcare, such as language barriers, lack of a support network, and difficulties accessing mental health services. However, facilitators were also identified, such as healthcare professionals taking the time to provide clear, respectful communication and openness toward discussing mental health in Belgium compared to their home countries. The study confirms prior insights on the complexity of perinatal care for migrant mothers, aligning the call to more culturally sensitive healthcare practices.

Not speaking the local language was the most reported barrier that hindered access to information. Although smartphone development has allowed continuous access to information about the perinatal period on digital media, information found might be of unequal quality, or professionals might overlook the women’s need for guidance^24^. Human sources are generally still considered more trustworthy^25^, albeit less accessible if there are language barriers or healthcare providers do not try to understand. In addition, increased use of digital health information depends on education level and increased digitalization of health services and information. This might further disadvantage those who do not have access, to begin with^25^. In addition, given the changes and risks related to digital health information, it might also warrant healthcare professionals to take a role in informing women or navigating them to trustworthy sources. The use of patient navigators is increasingly being asked for and has shown improvements in the uptake of referral and mental health treatment if found necessary^11^.

Our findings confirm difficulties reported in accessing mental healthcare during pregnancy among women of refugee background^11^. Stigma and cultural barriers were also reported here. For those who have not been accustomed to discussing mental health problems due to taboos in their home countries, overcoming this barrier becomes a critical consideration. Willingness to do so may be influenced by the fear of being perceived as abnormal, raising questions about the depth of these ingrained beliefs. The provision of early screening and accessible mental health services, as well as improved psycho-educational opportunities, are shown to alleviate perceived stress and enhance stress-coping abilities^26^. Stigma has a small- to moderate-sized negative effect on help-seeking^27^. For individuals who have not learned to discuss mental health issues due to taboos in their home countries, addressing these concerns becomes a complex task. The deeply ingrained cultural beliefs may affect the willingness to open up about such matters. One of the effects seemingly resulting from a higher level of mental health literacy is a decrease in stigma associated with mental illness. Stigma perpetuates negative attitudes and discriminatory actions, playing a detrimental role in the well-being of individuals with mental illness and creating considerable barriers to seeking care professionals, access to opportunities, and integration into the community^28^.

Due to their migration, exacerbated by the COVID-19 situation, most women expressed having a limited social network and being far from family, making it challenging to share the positive aspects of pregnancy and having a small baby with family members who may be in another country or are only accessible through video calls. Social support was often referred to as important and has been shown in other research as one of the protective factors for maternal anxiety and depression^29^. Including partners, if here, is therefore important. In addition, community-based befriending and peer support approaches might address women’s needs for social support^30^. In the study of Lebel et al.^31^, elevated symptoms of anxiety and depression were reported among pregnant individuals during the COVID-19 pandemic. Increased social support was associated with lower symptom burden^31^, and low social support increased postpartum depressive symptoms^32^.

While conducting the interviews, we noticed a vicious cycle of not speaking the language, not working, hence not engaging with people, lacking colleagues and friends, feeling lonely, and being unable to practice the language, which may further compound the challenges faced by the participants. This intersectionality has been reported repeatedly in other studies and policy reports as well^33,34^. The motivations for coming to Belgium may also influence how they perceive their current situation. Most participants relocated to Belgium due to unsafe and/or war-related conditions in their home countries. In addition, misinformation about legal rights and inadequate clarification during medical appointments frequently interacted with social determinants, such as low socioeconomic status, unemployment, and poor living conditions, resulting in lower perceived quality of healthcare. Special attention needs to be given to the most vulnerable populations to improve healthcare. Challenges reside not only in assuring access but also in promoting equity in the quality of care^28^. Integration of migrant status as a health determinant and having a specific focus on this during educational programs of nurses and midwives, is the first important step that will enhance professionals’ skills in assessing migrant status and understanding how this is complex and can contribute to barriers among migrant women accessing perinatal and mental health services^35^.

### Strengths and limitations

This study has several strengths. First, our diverse group of interviewees provided a broad perspective on the issue. We also took measures to ensure research reliability: 1) The interviewer critically examined the flow and questioning of interviews to identify errors and documented biases beforehand (bracketing); 2) Two researchers independently analyzed the data, comparing results for consistency; 3) A decision trail was maintained to document the rationale behind key decisions, mitigating confirmation biases; and 4) To enhance credibility, we focused on person, data, and research triangulation through maximum variation sampling^21^. We triangulated data from in-depth interviews with observational notes, including non-verbal communication and interview settings, such as the presence of children or partners. Two researchers independently analyzed the transcripts and discussed the findings with a third party. By interviewing participants from 6 weeks postpartum onwards, we explored effects during this critical period, mitigating recall bias^36^.

The findings of this study should be viewed in light of some limitations. First, all interviews were conducted in participants’ homes, with half involving the presence of children, family members, or partners, possibly limiting open communication. Second, although data saturation was achieved, the study focused on women from six different origin countries, not capturing the full diversity of migration backgrounds. Factors such as the specific region of origin and migration timing may have influenced their experiences with perinatal care in Belgium. Third, interpreters sometimes used different dialects, potentially altering nuances. Research predominantly conducted in English may miss information about certain groups. Also, the process of translation itself may have influenced the narrative flow. Additionally, participants were unfamiliar with the concept of peer support sessions, and even with explanations, the desired depth of information was not achieved. While most mothers reported not experiencing mental problems, four mentioned feeling down or having a bad period. We did not use scales like Edinburgh Postnatal Depression Scale (EPDS) or GAD-7 to explore (the risks of) anxiety or perinatal depression.

### Implications for practice and policy

To support the perinatal mental health of first-generation migrant women in Belgium, healthcare providers need training in cultural competence and health literacy capacity building and have to collaborate in a multidisciplinary matter to provide holistic care^37^. Strengthening social support networks through community-based programs and screening of mental health issues to lower barriers in help-seeking behaviors, can reduce isolation and enhance mental well-being^14^. Early intervention by a specialist midwife or other specialist care provider for antenatal follow-up has been called upon^38^. Policy efforts should focus on the intersectionality of the challenges regarding mental health and migration, reducing structural barriers, such as cost and accessibility, and improving associated psychosocial and labor circumstances for this group – factors shown to have a protective effect^39,40^.

## CONCLUSIONS

This study emphasizes the importance of a holistic approach that includes improved communication, enhanced social support, and structural healthcare changes to effectively address the unique needs of migrant mothers, ultimately improving their mental health and overall well-being. Findings highlight the urgent need for culturally and linguistically tailored perinatal mental health support. Healthcare systems must address barriers to ensure accessible and respectful care, enhancing mental health outcomes for migrant women. This requires awareness-raising and specific training for healthcare professionals, particularly midwives, to cater to the diverse needs of this population. Recognizing the large variation in this population is of paramount importance.

## Data Availability

The data supporting this research are available from the authors on reasonable request.
